# Phosphodiesterase-5 Inhibition Mimics Intermittent Reoxygenation and Improves Cardioprotection in the Hypoxic Myocardium

**DOI:** 10.1371/journal.pone.0027910

**Published:** 2011-11-28

**Authors:** Giuseppina Milano, Paola Bianciardi, Viviane Rochemont, Giuseppe Vassalli, Ludwig K. von Segesser, Antonio F. Corno, Marco Guazzi, Michele Samaja

**Affiliations:** 1 Centre Hospitalier Universitaire Vaudois, Lausanne, Switzerland; 2 Laboratorio di Biologia Vascolare e Medicina Regenerativa, Centro Cardiologico Monzino, IRCSS, Milan, Italy; 3 Department of Medicine, Surgery and Dentistry, University of Milan, Milan, Italy; 4 Cardiology, Fondazione Cardiocentro Ticino, Lugano, Switzerland; 5 Pediatric Cardiac Surgery, Prince Salman Heart Center, Riyadh, Kingdom of Saudi Arabia; Virginia Commonwealth University, United States of America

## Abstract

Although chronic hypoxia is a claimed myocardial risk factor reducing tolerance to ischemia/reperfusion (I/R), intermittent reoxygenation has beneficial effects and enhances heart tolerance to I/R. Aim of the study: To test the hypothesis that, by mimicking intermittent reoxygenation, selective inhibition of phosphodiesterase-5 activity improves ischemia tolerance during hypoxia. Adult male Sprague-Dawley rats were exposed to hypoxia for 15 days (10% O_2_) and treated with placebo, sildenafil (1.4 mg/kg/day, i. p.), intermittent reoxygenation (1 h/day exposure to room air) or both. Controls were normoxic hearts. To assess tolerance to I/R all hearts were subjected to 30-min regional ischemia by left anterior descending coronary artery ligation followed by 3 h-reperfusion. Whereas hypoxia depressed tolerance to I/R, both sildenafil and intermittent reoxygenation reduced the infarct size without exhibiting cumulative effects. The changes in myocardial cGMP, apoptosis (DNA fragmentation), caspase-3 activity (alternative marker for cardiomyocyte apoptosis), eNOS phosphorylation and Akt activity paralleled the changes in cardioprotection. However, the level of plasma nitrates and nitrites was higher in the sildenafil+intermittent reoxygenation than sildenafil and intermittent reoxygenation groups, whereas total eNOS and Akt proteins were unchanged throughout. Conclusions: Sildenafil administration has the potential to mimic the cardioprotective effects led by intermittent reoxygenation, thereby opening the possibility to treat patients unable to be reoxygenated through a pharmacological modulation of NO-dependent mechanisms.

## Introduction

Systemic hypoxia, or insufficient supply of O_2_ to tissues, is a common denominator in several diseases, including myocardial infarction, cyanotic congenital heart defects, pulmonary obstructive diseases and chronic cor pulmonale. Among these diseases, all of which reflect into systemic hypoxia, infants and children with congenital cyanotic heart defects represent a large population affected by the deleterious consequences of chronic systemic hypoxia. For many years, it was believed that the hypoxic state confers cardioprotection and renders hearts more tolerant to ischemia/reperfusion (I/R). However, it has been made clear that it is not hypoxia, but rather the reoxygenation after hypoxia, the real factor that confers cardioprotection. In facts, when exposed to chronic systemic hypoxia without reoxygenation, hearts exhibit marked deleterious alterations in several signaling paths, including K^+^_ATP_ channels [1], oxidative stress [2] and mitogen-activated protein kinases [3]. Such changes are followed by right ventricle dilatation with wall thickening [4], impaired tolerance to reoxygenation [2] and impaired ability to resist ischemia/reperfusion (I/R) injury [5]. These findings are corroborated by the clinical observation that the outcome of surgery aimed at repairing the cyanotic congenital heart defects is complicated by myocardial damage occurring because of the acute re-oxygenation at the moment of the institution of cardiopulmonary bypass with elevated oxygen content, followed by the I/R injury when heart is arrested to perform the intra-cardiac repair [6].

In principle, at least four treatments appear suitable to improve cardioprotection in chronically hypoxic patients: ischemic preconditioning, remote ischemic preconditioning, postconditioning and intermittent hypoxia. Actually, each of them presents features that prevent their full application in clinical contexts and the search for alternative treatments is imperative. In rats exposed to chronic systemic hypoxia for 2 weeks, daily reoxygenation events (or intermittent reoxygenation, IntReox) enable hearts to resist to I/R [5], perhaps as the result of modulation of some mitogen-activated kinases, namely phosphatidylinositol-3-kinase-protein kinase B (Akt) and extracellular signal-regulated kinases 1/2 (ERK1/2) [7]. Although this finding potentially addresses IntReox as an elective pretreatment to improve cardioprotection in hypoxic hearts, even IntReox is difficult to be applied in the clinical practice. Therefore, searching for pharmacological alternatives to IntReox may enable to devise a therapy whereby hypoxic patients benefit from IntReox without the need of the reoxygenation.

The NO/cyclic GMP (cGMP) pathway is a preponderant, although not exclusive, mechanism that mediates reoxygenation-induced cardioprotection [8]. The rationale is that the NO produced by endothelial cells activates smooth muscle cell soluble guanylate cyclase to produce cGMP that acts as a vasodilator and hence as a cardioprotective agent. As the intracellular level of cGMP is controlled by the activity of phosphodiesterase-5 (PDE5), it is expected that pharmacological inhibition of PDE5 by sildenafil might improve cardioprotection in the hypoxic myocardium. A pharmacological stimulator of ischemic preconditioning [9]–[11], sildenafil now represents a powerful therapeutical tool in several cardiovascular disorders [12]. Well tolerated for long-term treatment with few side-effects [13], sildenafil reduces pulmonary vascular resistance [14], improves arterial oxygenation in patients with pulmonary artery hypertension [15] and prevents altitude-induced hypoxemia [16], [17]. But the potential role of sildenafil to mimic the reoxygenation-induced cardioprotection during hypoxia is still unexplored.

This study aims at testing the hypothesis that sildenafil administration during systemic hypoxia mimics the beneficial effects led by IntReox. To this purpose, we will expose animals to chronic hypoxia for 2 weeks and compare the resistance to acute myocardial infarct in animals subjected to IntReox, sildenafil administration, or both.

## Materials and Methods

### Animals

A total of 109 male 5-week old Sprague-Dawley rats were exposed to hypoxia (10% O_2_) for 15 days in specially designed chambers that enable all kinds of treatments, including drugs administration and animals handling avoiding any exposure of the animals to atmospheric air [1], [2]. Rats divided into four groups: saline (n = 25), sildenafil (n = 22), IntReox (1-h normoxia per day, n = 19) and treated with both sildenafil and IntReox (n = 17). Of these, 6, 3 and 3 rats belonging to saline, sildenafil and IntReox groups, respectively, died on the first day of hypoxia. Therefore, final n was 19, 19, 22 and 17 rats in the four groups. Normoxic rats breathing room air in the same chambers used for hypoxia served as control (n = 20).

This study was carried out in strict accordance with the recommendations in the Guide for the Care and Use of Laboratory Animals of the National Institutes of Health. The protocol was approved by the Committee on the Ethics of Animal Experiments of the Centre Hospitalier Universitaire Vaudoise (Permit Number: 1325. 2). All surgery was performed under sodium pentobarbital anesthesia, and all efforts were made to minimize suffering.

Sildenafil was obtained from a 25-mg Viagra (Pfizer) tablet that was dissolved in 21.6 ml saline, filtered and stored at 4°C. This solution was given daily i. p. in the dose of 1.4 mg·kg^−1^ b. w. dissolved in 0.3 ml saline. Rats had free access to water and a laboratory diet until 24 h before sacrifice. For sacrifice, performed 24 h after the last aeration or administration of sildenafil, rats were anesthetized (100 mg·kg^−1^ thiopental and 1500 IU heparin i. p.) in the compensation chamber kept at 10% O_2_. The study was conducted in accordance with the *Guide for the Care and Use of Laboratory Animals*, published by the National Institutes of Health (NIH Publication No. 85-23, Revised 1996). Experimental protocols conformed to Swiss law and local ethical committee guidelines for animal research.

### LAD ligature

This part of the study was performed in 36 animals (7 normoxic and 29 hypoxia, including 7 saline, 8 treated with sildenafil, 8 IntReox and 6 sildenafil+IntReox). Anesthetized (100 mg·kg^−1^ sodium pentobarbital+500 IU heparin i. p.) rats were placed on a heating pad at 37°C and ventilated at 60 cycles·min^−1^ with a tidal volume of 2 ml (Harvard Apparatus model 683, Holliston, MA) with either room air or hypoxic atmosphere for normoxic and hypoxic groups, respectively. The left anterior descending coronary artery (LAD) was occluded for 30 min [18] and reperfused for 3 h, then the aorta was mounted on a cannula and perfused with 15–20 ml saline at room temperature to wash out the blood. LAD was re-occluded and saturated Evans blue (2 ml) injected to mark the ischemic zone as tissue area without the blue dye. The heart was frozen in liquid nitrogen and stored at −20°C until analysis.

To measure the infarct and risk areas, the frozen heart was cut into five/six 1-mm thick transverse slices from apex to base. The slices were incubated in triphenyltetrazolium chloride in sodium phosphate buffer at 37°C for 20 min to stain viable cells in the risk zone. Afterwards, the slices were immersed in 10% formalin for 4 days to enhance contrast between stained and unstained areas, with the latter representing the infarct size. The extent of stained and unstained areas was calculated for each slice from computerized images using NIH Image software (National Institutes of Health, USA). The risk area was expressed as percentage of total ventricle area whereas the infarct area was expressed as a percentage of the risk area.

### Biochemical analyses

This part of the study was performed in 27 animals (7 normoxic and 20 hypoxia, including 5 saline, 5 treated with sildenafil, 5 IntReox and 5 sildenafil+IntReox). Anesthetized rats were euthanized by removing heart and two blood samples were withdrawn into a heparinized tube to measure either hemoglobin concentration, hematocrit and red blood cell count (Abbott Cell-dyn 3500 R System, Baar, Switzerland) or plasma nitrates+nitrites (NOx) by the Griess reaction. The myocardium was quickly rinsed in ice-cold PBS (pH 7.4), clamped between steel tongs pre-cooled with liquid nitrogen and stored at −80°C until analysis.

The expression levels of eNOS and phosphorylated (P)-eNOS were measured in homogenized frozen tissue by Western blotting [18] using primary antibodies for eNOS (N-20: sc-653) and phosphorylated eNOS (Ser^1177^) (Santa Cruz Biotechnology) on a 8% denaturating gel, followed by incubation with HRP-conjugated secondary antibody. The same extract was used for the determination of Akt and P-Akt by phosphospecific antibodies against P-Akt (Ser^473^) (Cell Signaling Technologies). Band intensities were quantified by NIH AutoExtractor-1.51 software. The same extract from a normoxic heart was loaded on all blots for quantitative comparisons.

To measure cGMP, frozen tissue was homogenized at 4°C with 0.1 mol·l^−1^ HCl (10% wet wt/vol), centrifuged (2000 rpm for 10 min at 4°C) and cGMP measured using an immunoassay kit (Assay designs, Inc., MI). Caspase-3 activity was assayed by Caspase-3/CPP32 colorimetric assay kit (MBL).

### Immunofluorescence

Frozen biopsies were embedded in Optimum Cutting Temperature Compound (Leica Instruments, Nussloch, Germany), serial 5-µm thick sections were obtained in a cryomicrotome (Leica CM1510, Nussloch, Germany) and placed on silanized glass slides. Two distinct sections were obtained from each biopsy, and five fields were selected from each section. Care was taken in order to analyze the same areain all sections. The sections were dried (2 min at room temperature), fixed in 4% buffered formalin (45 min), rinsed 3 times in PBS (5 min/each), post-fixed with ethanol-acetic acid 2∶1 (vol∶vol) (5 min at −20°C), rinsed twice in PBS (5 min/each), boiled in 10 mM citrate at pH 6.0 (10 min), washed once in H_2_O and twice in PBS, and finally used for histochemistry.

To detect DNA fragmentation, we used the TUNEL ApopTag Red In Situ Apoptosis detection kit (Chemicon, International). The images were acquired and analyzed as described elsewhere [19], counting the number of TdT-positive nuclei by examining five random fields in a blind procedure. Values are expressed as the ratio (number of TdT-positive nuclei)/(total number of nuclei). The total number of nuclei was detected by Hoechst.

### Morphologic measurements

This part of study was performed in 32 animals (6 normoxic and 26 hypoxia, including 5 saline, 6 treated with sildenafil, 9 IntReox and 6 sildenafil+IntReox). For morphologic measurements, the hearts were excised, excess water was absorbed on tissue paper, and the heart mass was weighed. The atria were excised, and the free walls of the RV and LV and the septum were dissected free, dried for 48 hours at 90°C, and weighed separately. RV hypertrophy was assessed from the weight ratio of the RV to LV plus interventricular septum (IS) (RV/[LV+IS]).

### Statistics

Data are expressed as mean±SEM. Significance level was P = 0.05 (two-tailed). To detect differences among the groups, we routinely performed one-way ANOVA. If this test resulted significant, the differences between selected pairs of data were tested using the Bonferroni procedure. Time courses were analyzed and compared by the Mann-Whitney test.

## Results

### Body weight, cardiac hypertrophy and polycythemia

The weight of the animals after thetreatments was less in all hypoxic groups compared to normoxia (Table 1). Within the hypoxic groups, the various treatments attenuated, without eliminating, the effect of hypoxia on body weight. Myocardial hypertrophy, assessed from the heart/body weight ratio, increased in hypoxic saline-treated rats (P<0.001 vs. normoxia). Whereas the treatment with sildenafil attenuated such increase (P<0.001 sildenafil vs. saline), IntReox was ineffective (P = NS vs saline). When the two treatments were combined, the heart/body weight ratio returned to the normoxic value (P<0.001 vs. both saline-treated and IntReox animals). Hematocrit, blood hemoglobin concentration and red blood cell count were elevated in hypoxic animals with respect to normoxic (P<0.0001 for all the parameters). Both treatments were ineffective.

**Table 1:** Animal and hematological measurements.

	Normoxia	Chronic hypoxia			
		Saline	Sildenafil	IntReox	Sildenafil+IntReox
Initial body weight, g	251±2	248±5	255±9	250±5	235±6
Gain in body weight, g	98±7	−4±10*	73±21#	16±4$	25±5#
Heart/body weight, mg·g^−1^	3.76±0.11	5.57±0.22*	4.28±0.21#	5.21±0.09$	3.75±0.16#
Hematocrit	0.51±0.02	0.66±0.02*	0.65±0.01	0.68±0.01	0.62±0.01
Hemoglobin, g·l^−1^	158±4	200±7*	202±5	218±3	205±4
Red blood cell count, RBC·fl^−1^	8.65±0.36	10.45±0.43*	10.05±0.29	9.80±0.19	9.40±0.18

*, P<0.05 when comparing untreated chronic hypoxia vs. normoxia;

#, P<0.05 when comparing the various treatments vs. untreated chronic hypoxia;

$, P<0.05 when comparing the two treatments between them. All post-tests performed with the Bonferroni method.

Data are mean±SEM.

ANOVA P<0.0001 for all measurements.

### Tolerance to acute infarct

**Figure 1:**
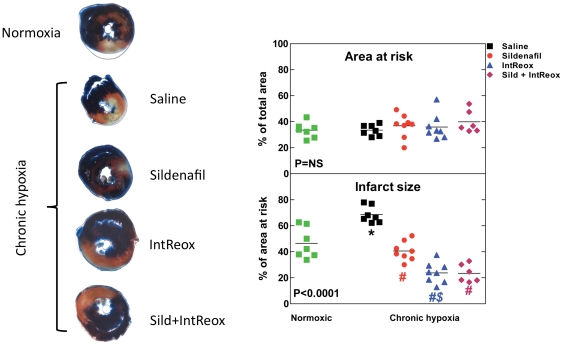
Tolerance to acute infarct. Hearts were subjected to 30-min occlusion of the left anterior descending coronary artery followed by 3-h reperfusion, and were stained as described in Materials and Methods. The ***left panel*** shows sample photographs of the central sections of one heart per group. The ***right panel*** shows average area at risk (top) and infarct size (bottom) along withvalues obtained in single experiments. The P value in the insets reports ANOVA. *, P<0.05 when comparing untreated chronic hypoxia vs. normoxia; #, P<0.05 when comparing the various treatments vs. untreated chronic hypoxia; $, P<0.05 when comparing the two treatments between them. All post-tests performed with the Bonferroni method.

Figure 1 shows the effects of the various treatments on the risk area and the infarct size after LAD ligation for 30 min and reperfusion for 3 h. When expressed as percentage of total ventricular area, the area at risk was the same in all the groups (ANOVA P = NS). Thus, the stress imposed to the heart had the same severity in all the groups. However, the infarct size, expressed as percentage of the area at risk (ANOVA P = 0.001), was markedly increased in hypoxic with respect to normoxic hearts (68.5±2.4% vs. 46.5±4.4%, P = 0.001). *In vivo* administration of sildenafil reduced the infarct size to 40.6±2.6% of the area at risk (P = 0.001). IntReox animals displayed reduced infarct size to 23.8±2.8% of the area at risk (P = 0.001). The reduction afforded by IntReox was greater than that afforded by the treatment with sildenafil (P = 0.01). When animals were treated with both sildenafil and IntReox, the resulting pattern was the same as that observed in IntReox hearts, with the infarct size (23.5±2.8% of the area at risk) markedly reduced with respect to both hypoxia (P = 0.001) and sildenafil (P = 0.01). Thus, both sildenafil administration and IntReox protect from I/R injury, and the combination of the two treatments apparently does not yield any additional synergistic protection.

### Apoptosis

**Figure 2:**
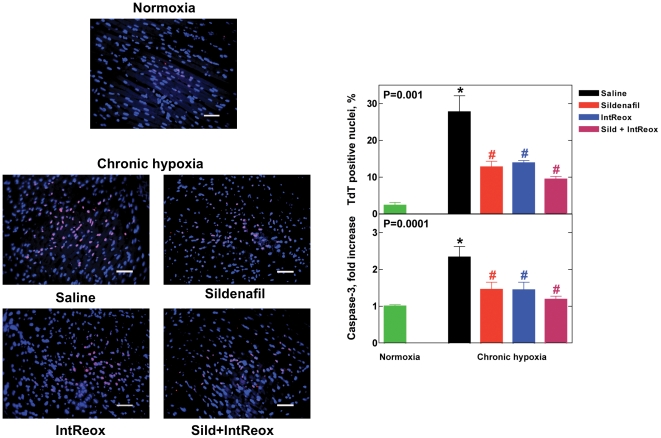
Apoptosis. The ***left panel*** shows representative immunofluorescence images from the various groups under study. TdT-positive nuclei are marked in red, whereas total nuclei are marked in blue. Overlap red+blue gives pink. Magnification 40×, the bar represents 20 µm. ***Right top panel***. ***The*** left axis reports the percent of TdT-positive/total nuclei in all the samples (five sections per each biopsy, and five fields per each section). In the ***Right bottom panel***, the activation of caspase-3 activity is reported. The P value in the insets reports ANOVA. Sample photographs of the central sections of one heart per group are shown. *, P<0.05 when comparing untreated chronic hypoxia vs. normoxia; #, P<0.05 when comparing the various treatments vs. untreated chronic hypoxia; $, P<0.05 when comparing the two treatments between them. All post-tests performed with the Bonferroni method.

We assessed this phenotype by measuring the extent of DNA fragmentation by the TUNEL assay and the activation of caspase-3 (Figure 2). For both parameters, untreated chronic hypoxia increased the degree of apoptosis, whereas all treatments attenuated the effects of hypoxia by reducing the degree of apoptosis. In no case, however, the effects of hypoxia were completely reversed. Thus, both treatments enabled control of hypoxia-induced apoptosis, but without additional synergistic effects.

### Biochemical analyses

**Figure 3:**
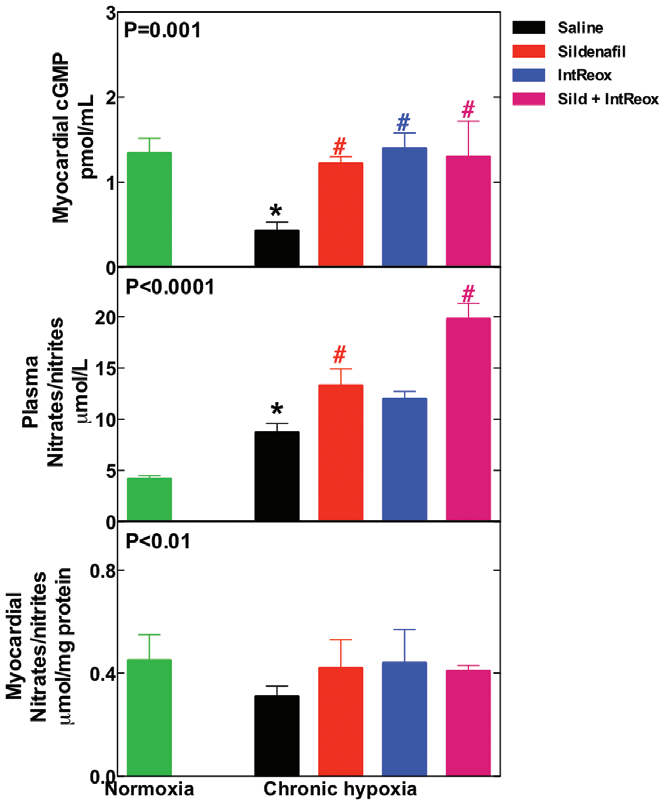
Effects of NO. Myocardial level of cGMP (top panel), plasma level of nitrates/nitrites (middle panel) and myocardial nitrates/nitrites level (botton panel). The P value in the insets reports ANOVA. *, P<0.05 when comparing untreated chronic hypoxia vs. normoxia; #, P<0.05 when comparing the various treatments vs. untreated chronic hypoxia. All post-tests performed with the Bonferroni method.

The myocardial cGMP level was depressed by hypoxia with respect to normoxia (0.43±0.10 pmol·ml^−1^ vs. 1.20±0.31 pmol·ml^−1^, respectively, P = 0.01, Figure 3, top panel). Both *in vivo* administration of sildenafil and IntReox restored the cGMP level. The combined treatments did not further increase cGMP. By contrast, the myocardial NOx level (Figure 3, bottom panel) remained unaffected by the treatments.

The plasma level of NOx, an index of NO production, increased markedly during hypoxia from 4.5±1.0 µmol·l^−1^ to 8.7±0.9 µmol·l^−1^ (P = 0.001, Figure 3, middle panel). *In vivo* administration of sildenafil further increased NOx (P = 0.05). By contrast, IntReox did not cause a significant increase of NOx with respect to saline-treated animals. Combining sildenafil with IntReox led to a substantially higher NOx (P = 0.001 vs. sildenafil and aeration). Thus, whereas hypoxia increased NO production, sildenafil enabled further increase of this parameter, but IntReox alone could not increase appreciably NOx.

### Nitric oxide synthase

**Figure 4:**
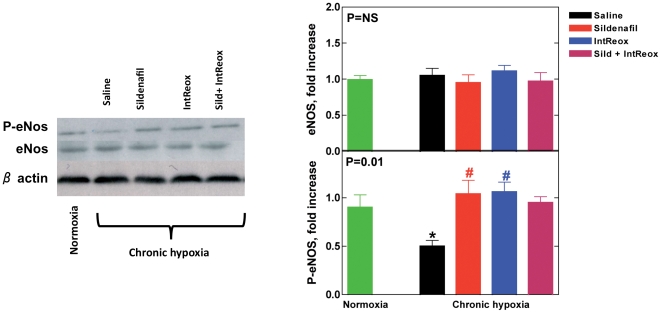
eNOS activity. The ***left panel*** shows representative Western blots performed using antibodies against total eNOS protein and phosphorylated eNOS, with β-actin as loading control. All blots were subjected to densitometry and the ***right panel*** shows the average expression of total eNOS (top) and phosphorylated eNOS (bottom). The P value in the insets reports ANOVA. *, P<0.05 when comparing untreated chronic hypoxia vs. normoxia; #, P<0.05 when comparing the various treatments vs. untreated chronic hypoxia. All post-tests performed with the Bonferroni method.

To address the expression level of eNOS and its phosphorylation, Figure 4, top panel, shows that eNOS expression was unaffected by hypoxia as well as the various treatments. By contrast, phosphorylated eNOS was decreased in hypoxic with respect to normoxic hearts (P = 0.05, Figure 4, bottom panel). Both treatments restored the normoxic eNOS phosphorylation (P = 0.05). Despite a slight reversal of the effect led by sildenafil and IntReox, their combination did not change significantly the pattern (P = NS). Thus, both treatments enhanced eNOS phosphorylation, but without effects on eNOS expression.

### Akt signaling

**Figure 5:**
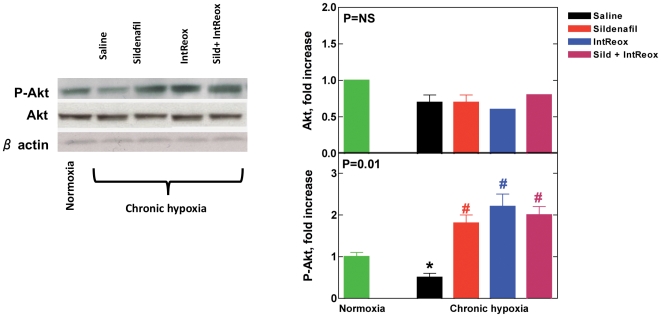
Akt activity. The ***left panel*** shows representative Western blots performed using antibodies against total Akt protein and phosphorylated Akt, with β-actin as loading control. All blots were subjected to densitometry and the ***right panel*** shows the average expression of total Akt (top) and phosphorylated Akt (bottom). The P value in the insets reports ANOVA. *, P<0.05 when comparing untreated chronic hypoxia vs. normoxia; #, P<0.05 when comparing the various treatments vs. untreated chronic hypoxia. All post-tests performed with the Bonferroni method.

Figure 5, top panel, shows that, like eNOS expression, total Akt expression was unaffected by neither hypoxia nor the various treatments. By contrast, phosphorylated Akt decreased in hypoxic with respect to normoxic hearts (P = 0.0001). Both treatments led to overshooting P-Akt (P = 0.0001), with no additional effect due to the combination of the treatments (P = NS).

## Discussion

Exposure of animals to chronic systemic hypoxia for 15 days depressed their resistance to acute myocardial infarct. Either IntReox or administration of sildenafil during hypoxia reverted such deleterious effect without affecting polycythemia and with negligible effects of animal homeostasis. This phenotype was paralleled by changes in apoptosis, myocardial cGMP, P-Akt and P-eNOS. No cumulative effect of the combination of the treatments was observed, with the exception of plasma NOx. As both the reoxygenation and sildenafil increased plasma NOx, myocardial cGMP and eNOS Ser^1177^ phosphorylation, the up-regulation of the NO/cGMP signaling pathway appears to be a critical factor to enhance tolerance to acute myocardial infarct during hypoxia.

### Sildenafil-induced cardioprotection

A key issue in our study, the hypoxia-induced impairment of cardioprotection confirms previously reported data [5]. Likewise, IntReox has the potential to improve myocardial tolerance to I/R, and is mimicked by treatment with sildenafil.

Although several studies have focused into the role of PDE5 inhibition on I/R injury in normoxia [10], [20]–[22], this is to our knowledge the first report demonstrating that sildenafil administration during systemic hypoxia is cardioprotective. We tested a single sildenafil dose within the range that saturates protection in humans [23]. Despite the plasma half-life of 4–6 h [24], the selected dose substantially elevated myocardial cGMP, indicative of efficient inhibition of PDE5 activity. High cGMP leads to acute and delayed cardioprotection via mitochondrial K^+^_ATP_ channels opening [25] and mediates preconditioning in rat hearts [11]. In a normoxic model of I/R, sildenafil was shown to induce delayed preconditioning by elevating iNOS and eNOS mRNA and proteins levels [26]. We didn't observe any elevation in eNOS, but the difference in the experimental models, acute vs. chronic hypoxia, may account for this discrepancy. Furthermore, our data are consistent with constant eNOS, as opposed to increased P-eNOS, in chronically hypoxic mice irrespectively of sildenafil [27]. In a model of rabbit hypoxic from birth that involved myocardial reoxygenation, eNOS phosphorylation at Ser^1177^ increased [28], consistently with our data that show greater P-eNOS in reoxygenated than hypoxic hearts. Although eNOS can be phosphorylated in multiple sites, its phosphorylation at Ser^1177^, as probed in this study, indicates the effective degree of electron flow through eNOS [29].

Apoptosis is crucial in determining cardioprotection. Chronic hypoxia enhances cardiomyocytes apoptosis [30], in part through stress-activated protein kinases/c-Jun NH_2_-terminal kinases 1 and 2 (JNKs) and p38 MAPK [27]. Here, PDE5 inhibitiondecreased pro-apoptotic caspase-3 and DNA fragmentation, in agreement with work documenting that PDE5 inhibition modulates the pathways operating through p38, ERK1/2 and NO [26], [31]. It is unlikely, however, that increased apoptosis represents the only cause for worse tolerance to ischemia, because IntReox animals treated with sildenafil failed to further decrease apoptosis, yet the infarct size was less than in either IntReox and sildenafil-treated animals.

### Reoxygenation-induced cardioprotection

The finding that IntReox is protective against I/R addresses the relevance of the paths activated by sildenafil to induce cardioprotection. Up-regulation of cGMP in the absence of PDE5 inhibition points to NO-driven stimulation of soluble guanylate cyclase as regulator of intracellular cGMP in addition to PDE5 inhibition. Perhaps IntReox enhances the expression of some isoforms of NO synthase in endothelial cells, which escapes the methods used and is out of the aims of this study. However, the effects of IntReox and sildenafil were not cumulative, probably because the system was already saturated by either treatment.

A matter of concern in Figure 3, plasma NOx was increased in Sild+IntReox with respect to Sildenafil and IntReox, whereas myocardial cGMP remained constant. First, the occurrence of a linear relationship between plasma NOx and myocardial cGMP is unlikely. Second, the possibility that cell cGMP levels off at approximately 1–1.5 pmol/ml can't be ruled out as this value was never trespassed in our experiments. Indeed, even when sildenafil was administered in normoxic rats (experiments not shown), cGMP remained constant (1.34±0.18 vs. 1.35±0.21 pmol/ml, P = NS) despite increased plasma NOx from 4.5±0.1 to 7.79±1.06 µM (P<0.001). The myocardial NOx level was insensitive to the various experimental conditions probably because of fast scavenging of intracellular NO by NO-binding proteins.

As Akt blockade with LY-294002 reverted the protection afforded by IntReox [7], we verified whether the mechanisms underlying protection in sildenafil and IntReox groups share similar pathways, and found similar changes in P-Akt in both groups.

The delayed cardioprotective mechanism linked with IntReox might share several features with those related to intermittent hypoxia [32]. The potentially involved mechanisms include up-regulation of NO production by eNOS [33] and down-regulation of intracellular calcium by mitochondrial K^+^_ATP_ channels opening [34], [35] (but a report shows that K^+^_ATP_ channels are not responsible for increased tolerance after hypoxia [36]). Furthermore, transcriptional and/or post-translational changes in endoplasmic reticulum calcium cycling proteins might occur [37], [38], as well as increased expression of vascular endothelial growth factor and its receptors [39], which may lead to the control of apoptosis with improved cell viability [40]. IntReox might recruit analogous mechanisms. In facts, IntReox has been shown to recover heart K^+^_ATP_ channels [1] and mitogen-activated protein kinases [3], as well as skeletal muscle immunoreactivity to heat-shock protein-70, heme-oxygenase-1 [41] and cytochrome c oxidase [42]. These features would in principle lead to cardioprotection, independently of polycythemia and cardiomyocyte morphology [4].

### Conclusion

Exposure to chronic systemic hypoxia for 15 days markedly depresses myocardial tolerance to acute myocardial infarct. Either inhibition of PDE5 by sildenafil during hypoxia or IntReox attenuate this effect, but the mechanisms do not appear to be cumulative. The observed patterns are associated with improved NO metabolism and reduced activation of apoptotic pathways. Thus, *in vivo* sildenafil treatments may have a good potential application in targeting systemic hypoxia-induced derangement of cardiovascular function.
