# Correction: Phosphodiesterase-5 Inhibition Mimics Intermittent Reoxygenation and Improves Cardioprotection in the Hypoxic Myocardium

**DOI:** 10.1371/annotation/f7aebee7-7b7e-4a44-aa23-56e9a32f14f6

**Published:** 2012-01-23

**Authors:** Giuseppina Milano, Paola Bianciardi, Viviane Rochemont, Giuseppe Vassalli, Ludwig K. von Segesser, Antonio F. Corno, Marco Guazzi, Michele Samaja

There was an error in the affiliation designation of author Marco Guazzi. Marco Guazzi's correct affiliation is: 3 Department of Medicine, Surgery and Dentistry, University of Milan, Milan, Italy 

